# Insulin Growth Factor 1 Receptor Expression Is Associated with *NOTCH1* Mutation, Trisomy 12 and Aggressive Clinical Course in Chronic Lymphocytic Leukaemia

**DOI:** 10.1371/journal.pone.0118801

**Published:** 2015-03-18

**Authors:** Francesco Maura, Laura Mosca, Sonia Fabris, Giovanna Cutrona, Serena Matis, Marta Lionetti, Luca Agnelli, Marzia Barbieri, Marianna D’Anca, Martina Manzoni, Monica Colombo, Carlotta Massucco, Daniele Reverberi, Massimo Gentile, Anna Grazia Recchia, Sabrina Bossio, Fiorella Ilariucci, Caterina Musolino, Francesco Di Raimondo, Agostino Cortelezzi, Fortunato Morabito, Manlio Ferrarini, Antonino Neri

**Affiliations:** 1 Department of Clinical Sciences and Community Health, University of Milano and Hematology 1 CTMO, Foundation IRCCS Ca’ Granda, Ospedale Maggiore Policlinico, Milano, Italy; 2 SS Molecular Diagnostics, IRCCS S. Martino-IST, Genova, Italy; 3 Scientific Direction IRCCS S. Martino-IST, Genova, Italy; 4 Department of Pathology IRCCS S. Martino-IST, Genova, Italy; 5 U.O.C. di Ematologia, Azienda Ospedaliera di Cosenza, Cosenza, Italy; 6 Division of Hematology, A.O. Arcispedale Santa Maria Nuova-IRCCS, Reggio Emilia, Italy; 7 Division of Haematology, University of Messina, Messina, Italy; 8 Department of Biomedical Sciences, Division of Hematology, University of Catania and Ferrarotto Hospital, Catania, Italy; University of Sydney, AUSTRALIA

## Abstract

*IGF1R* is emerging as an important gene in the pathogenesis of many solid and haematological cancers and its over-expression has been reported as frequently associated with aggressive disease and chemotherapy resistance. In this study we performed an investigation of the role of *IGF1R* expression in a large and representative prospective series of 217 chronic lymphocytic leukaemia (CLL) patients enrolled in the multicentre O-CLL1 protocol (clinicaltrial.gov #NCT00917540). High *IGF1R* gene expression was significantly associated with *IGHV* unmutated (*IGHV*-UM) status (p<0.0001), high CD38 expression (p<0.0001), trisomy 12 (p<0.0001), and del(11)(q23) (p=0.014). Interestingly, higher *IGF1R* expression (p=0.002) characterized patients with *NOTCH1* mutation (c.7541_7542delCT), identified in 15.5% of cases of our series by next generation sequencing and ARMS-PCR. Furthermore, *IGF1R* expression has been proven as an independent prognostic factor associated with time to first treatment in our CLL prospective cohort. These data suggest that *IGF1R* may play an important role in CLL biology, in particular in aggressive CLL clones characterized by *IGHV*-UM, trisomy 12 and *NOTCH1* mutation.

## Introduction

Chronic lymphocytic leukaemia (CLL) is a common lymphoproliferative disorder characterized by heterogeneous clinical course. During recent years a number of prognostic markers have been proposed in order to distinguish between aggressive and indolent disease [1–3]. The mutational status of the immunoglobulin heavy chain variable region (*IGHV*) represents one of the most important prognostic markers in CLL and defines two disease subgroups: the first, characterized by the absence of *IGHV* somatic mutation (*IGHV*-UM) in CLL cells, is associated with the worst clinical course and outcome; the second presents *IGHV* somatic mutations (*IGHV*-M) and has a more favorable prognosis and outcome [4,5]. However, a significant fraction of *IGHV*-M patients also progresses and requires treatment, a finding that prompts the identification of novel putative clinical and biological markers able to stratify progression risk among these patients.

Insulin Growth Factor 1 receptor (*IGF1R*) is emerging as an important gene involved in many solid and haematological cancers. Higher expression of the gene has been associated with more aggressive disease and with pharmacological resistance [6]. Specifically the IGF1R-IGF1/2 interaction is involved in the constitutive activation of many important intracellular cell signalling pathways such as RAS/RAF/MEK/ERK kinases that stimulate cellular proliferation, or the phosphoinositide-3 kinase (PI3K)-Akt/mammalian target of rapamycin (mTOR) pathway that predominantly mediates cell survival. Recently *IGF1R* was reported to be correlated with the NOTCH1 signalling pathway in T-lymphoblastic leukemia where *NOTCH1* activating mutations occur in more than half of patients [7]. In addition, the *IGF1R* was recently tested as novel therapeutic target in solid malignancies and in multiple myeloma where *IGF1R* inhibitors appear to overcome bortezomib resistance in the malignant plasma cell [8,9].

Although it has been described that *IGF1R* is heterogeneously expressed in CLL [10], its role in the disease remains to be fully elucidated. Recently it was shown that IGF1R was generally over-expressed in CLL compared to healthy B-cells, and was implicated in the activation of the PI3K/Akt and MAPK pathways; notably, distinct CLL groups [i.e del(11)(q13), del(17)(p13) and trisomy 12 patients] were found to be associated with a significant higher *IGF1R* expression compared to other patients [11].

Here we investigate the relevance of *IGF1R* mRNA expression in a large and representative prospective series of Binet stage A CLL patients. In particular, we addressed its possible correlations with biological, molecular and clinical outcome.

## Materials and Methods

### Patients

Newly diagnosed Binet stage A CLL patients from several Italian Institutions were prospectively enrolled into the O-CLL1 protocol (clinicaltrial.gov #NCT00917540). Written informed consent was obtained from all patients in accordance with the declaration of Helsinki and the study was approved by the local Ethics Review Committee (Comitato Etico Provinciale, Modena, Italy). Diagnosis was confirmed by the biological review committee according to flow cytometry analysis centralized at the National Cancer Institute (IST) as previously described [12]. Treatment was decided uniformly in all participating centres based on documented progressive and symptomatic disease according to NCI-Working Group guidelines [2]. To date, 463 Binet A CLL patients have been selected according to the inclusion criteria of the protocol. Two hundred and seventeen CLL patients for whom RNA material was available were analysed by means of GeneChip Gene 1.0 ST Array (Affymetrix) as previously described [12] and *IGF1R* gene expression data have been inferred for each case. Gene expression data has been deposited in the National Centre for Biotechnology Information’s Gene Expression Omnibus database http://www.ncbi.nlm.mih.gov/geo and are accessible through series accession number GSE51529. Biological and molecular analyses were performed in peripheral CD19+ B-cell samples collected from all patients within 12 months after diagnosis as previously described [13,14].

CD38 expression was investigated as previously described and a 20% cut-off was chosen to discriminate positive from negative patients [15]. Fluorescence in situ hybridization (FISH) analyses of the most common cytogenetic deletions, *IGHV* mutational status and stereotyped *HCDR3* analysis were performed as previously described [16,17]. *NOTCH1* c.7541_7542delCT mutation was tested in 199 cases by next generation sequencing using the Roche 454 technology and subsequently validated by Amplification Refractory Mutation System (ARMS)-PCR as previously described [18].

According to the 1996 NCI guidelines [19], 162 (74.7%), 40 (18.4%) and 15 (6.9%) patients were classified as Rai 0, Rai I and Rai II, respectively. However, based on the more recent International Workshop on Chronic Lymphocytic Leukaemia (IWCLL) diagnostic criteria [2], 45 (20.7%) Rai stage 0 patients having less than 5.0x10^9^/L circulating monoclonal B lymphocytes were reclassified as clinical monoclonal B-lymphocytosis (cMBL). Patients’ clinical and biological profiles are summarized in Table 1.

**Table 1:** Clinical and biological features of patients enrolled in the O-CLL1 protocol and investigated by gene expression profiling.

	All pts (217)	MBL (45)	CLL (172)
**Median Monoclonal Lymphocytes**	7789 × 10^6^/L	3568 × 10^6^/L	9152 × 10^6^/L
**CD38>20%**	49/217 (26%)	12/45 (28%)	50/172 (29%)
***IGHV*-UM**	84/216 (39%)	16/45 (35%)	68/172 (39.5%)
**Normal Cytogenetic**	79 (36.4%)	14/45 (31%)	65/172 (37%)
del(13)(q14)*	88 (40.6%)	17/45 (37%)	70/172 (41%)
**Trisomy 12**	28 (12.9%)	13/45 (29%)	15/172 (9%)
**del(11)(q23)**	16 (7.4%)	1/45 (2.2%)	16/172 (9.2%)
**del(17)(p13)**	6 (2.7%)	0/45 (0%)	6/172 (
***NOTCH1* mutation**	31/199 (15.5%)	8/38 (19%)	23/161

*As sole genomic aberrations

### Real-Time Quantitative Polymerase Chain Reaction

*IGF1R* gene expression levels were analysed in purified CLL CD19+ cells by means of quantitative real-time polymerase chain reaction (Q-RT-PCR) assay. Total RNA was converted to cDNA using M-MLV reverse transcriptase (Invitrogen). Inventoried TaqMan Gene Expression Assay (Hs00609566_m1) and the TaqMan Fast Universal Master Mix were used according to manufacturer’s instructions (Applied Biosystems, Foster City, CA). 18S TaqMan Gene Expression Control (Hs99999901_s1) (Applied Biosystems) was used as the internal control. The measurement of transcript expression was performed using the Applied Biosystems StepONE Real-Time PCR System. All the samples were run in duplicate. Data were expressed as 2^-ΔCt^ (Applied User Bulletin No. 2).

### Statistical Analysis

All contingency analyses were performed by Fisher’s exact test. Quantitative variables were compared using the Mann-Whitney test. Time to First Treatment (TTFT) was defined as time from diagnosis to first line treatment. Cox proportional hazards model was used in the global test function of R software (under 100,000 permutation) to test the positive or negative association between the *IGF1R* expression levels (as covariate, assumed as continuous variable) and clinical outcome (as response variable, in terms of TTFT). Kaplan-Meier method was used for TTFT curve estimation. Cox proportional hazard model was used for multivariate analysis. *P*<0.05 was considered significant for all statistical calculations. All the analyses were performed using appropriate functions in R software (www.r-project.org).

## Results

We assessed the expression levels of the *IGF1R* gene in 217 cases included in a prospective cohort of Binet-A CLL patients. *IGF1R* expression showed a heterogeneous distribution, but no difference was found between cMBL and other CLLs or Rai 0 patients (data not shown). The levels of *IGF1R* transcripts were also validated by means of Q-RT-PCR in all samples for which RNA was available (61 cases), and showed very good concordance with microarray data (Pearson correlation, *r* = 0.82) (S1 Fig.).

**Fig 1:**
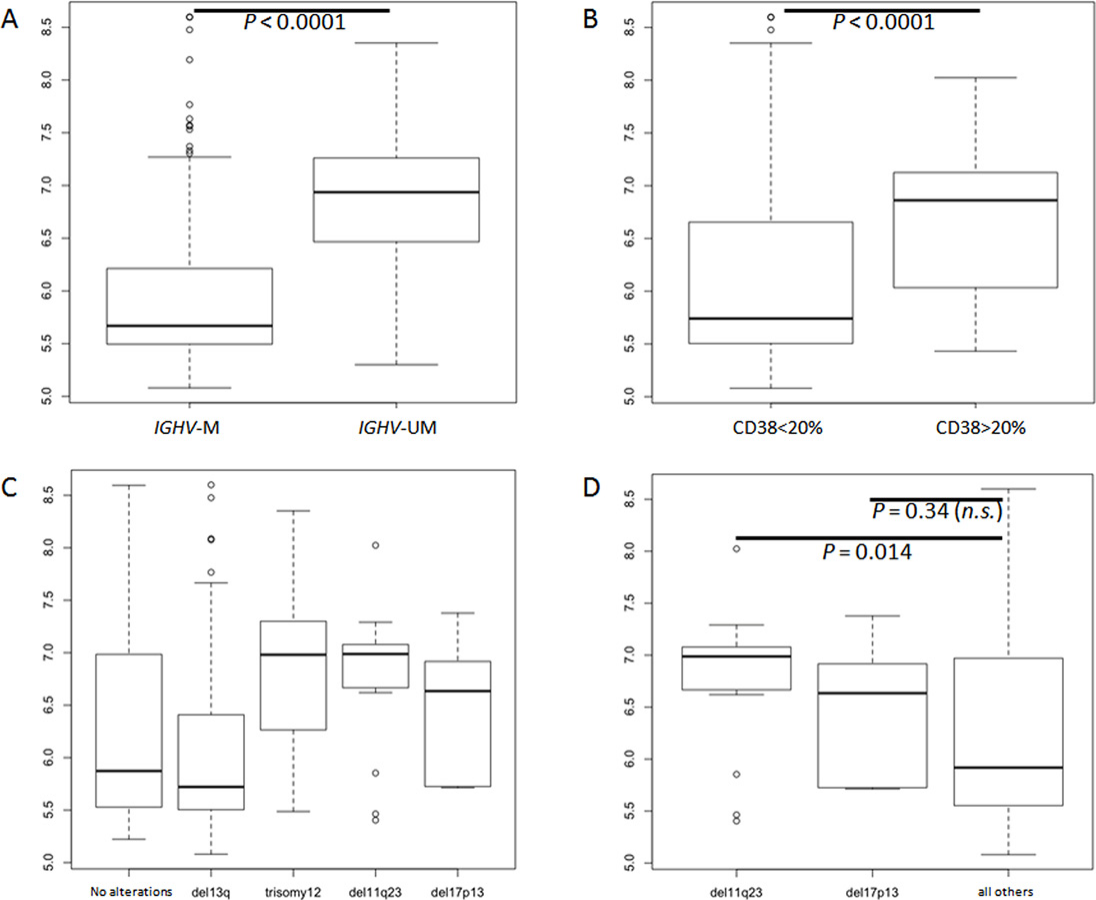
Boxplot of IGF1R expression (217 pts) in: A) *IGHV*-M vs *IGHV*-UM; B) CD38<20% vs CD38>20%; C) Most common cytogenetic aberrations evaluated by FISH; D) unfavorable cytogenetic deletions (del11q23 and del17p13) vs other patients (favorable/intermediate FISH).

Interestingly among 217 tested patients, *IGHV*-UM cases were characterized by significantly higher *IGF1R* expression compared to *IGHV*-M cases (p<0.0001) (Fig. 1A). This difference was confirmed also if cMBL and CLL patients were considered separately (p = 0.004 and p<0.0001, respectively). Stereotyped HCDR3 was identified in 70 cases (32%) (S1 Table), mainly characterized by *IGHV*-UM configuration (p<0.0001). Patients with stereotyped HCDR3 sequences showed greater *IGF1R* expression compared to non-stereotyped HCDR3 (p = 0.001); however, this could in all likelihood be related to the higher frequency of *IGHV*-UM among stereotyped HCDR patients [45/70; 64%]. Among most frequent stereotyped subset we observed a significant *IGF1R* overexpression among subset #1, #7, #8 and #10. These last two stereotyped subsets were known to be strictly associated with *IGHV*-UM, *IGHV4–39* utilization and trisomy 12. Of note, subset #4 patients (known to exhibit an indolent clinical course, unique *IGHV*-M and *IGHV4–34* utilization and distinct biological profile [17,20]) were characterized by lower *IGF1R* expression in all but one (1/10). This patient was the only one who progressed and required treatment among subset #4 subtype. Remarkably, *IGF1R* expression differed significantly between subset #4 patients and *IGHV*-M patients (p<0.0001 and p = 0.03 respectively) (S2 Fig.).

**Fig 2:**
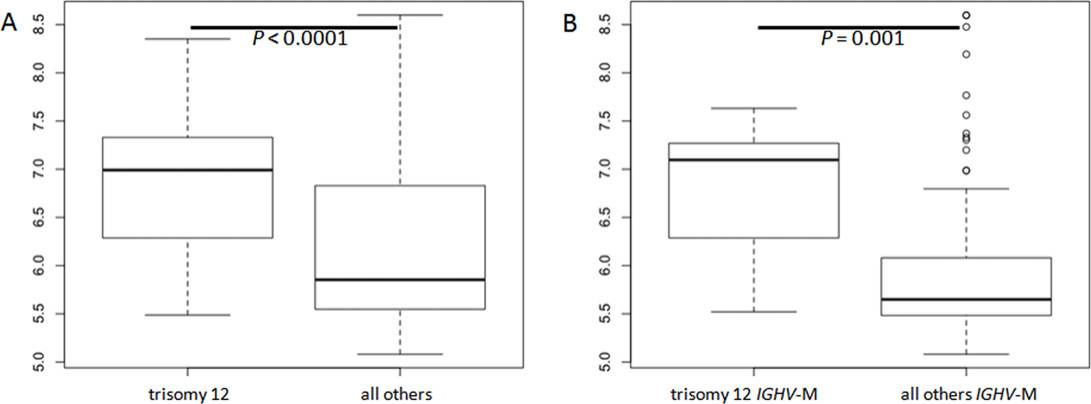
Boxplot of IGF1R expression in: A) Trisomy 12 vs no trisomy 12 patients; and B) Trisomy 12 vs no trisomy 12 considering only *IGHV*-M patients.

Patients with CD38 positive expression were generally characterized by higher *IGF1R* expression in almost all cases (p<0.0001) (Fig. 1B). This significant association was confirmed also if 30% was considered as CD38 positivity cut-off (data not shown). Higher *IGF1R* expression was also associated with unfavorable cytogenetic deletion [i.e. del(11)(q23) and del(17)(p13)], particularly with del(11)(q23) (p = 0.014) (Fig. 1C-D). The association between unfavorable cytogenetic deletions and *IGF1R* expression was not tested among cMBL due to the lower prevalence of those aberrations in that subgroup of patients. However, cases with del(13)(q14) as unique lesion, were characterized by the lowest *IGF1R* gene levels considering either all patients (Fig. 1C) and cMBL or CLL separately (p = 0.0004 and p = 0.001, respectively). On the contrary, among the most common cytogenetic aberrations, trisomy 12 showed stronger *IGF1R* expression compared with the other patients (p<0.0001) (Fig. 2A). *IGF1R* was highly expressed in almost all patients with trisomy 12 and this association was independent both from cMBL/CLL classification and from *IGHV* mutational status. In fact considering only *IGHV*-M patients, the presence of trisomy 12 was associated with a significantly higher *IGF1R* expression compared to the others (p = 0.001) (Fig. 2B).

**Fig 3:**
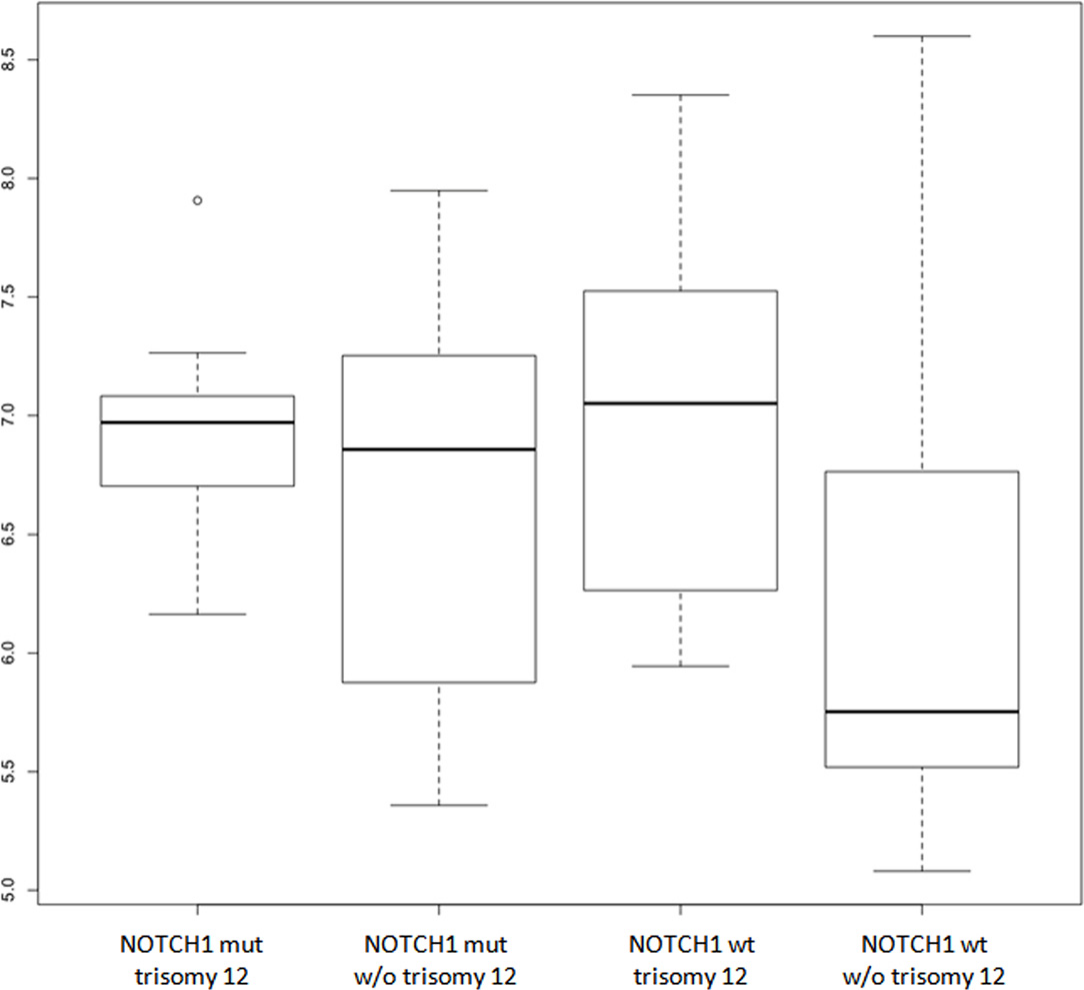
Boxplot of *IGF1R* expression in patients stratified according to trisomy 12 and NOTCH1 mutation (mut: mutation; wt: wild-type; w/o: without). *IGF1R* was significant higher among the NOTCH1 mut/trisomy12, NOTCH1 mut/NOtrisomy12 and NOTCH1,wd/trisomy12 compared to other patients (p = 0.0035, p = 0.0031 and p = 0.0002 respectively).

*NOTCH1* c.7541_7542delCT mutation was previously investigated by next generation sequencing Roche 454 technology in 199 (91.7%) patients included in the present study [18]. We considered as mutated only those patients (31 cases; 15.5%) in whom the presence of the dinucleotide deletion was confirmed by ARMS-PCR [18]. In our present series, *NOTCH1* mutation was confirmed to be strongly associated with trisomy 12, I*GHV4*–39 gene usage and with shorter TTFT in univariate analysis, whereas no difference in *NOTCH1* mutation prevalence was observed comparing cMBL, with CLL or Rai 0 patients (data not shown). Interestingly, patients with *NOTCH1* mutation showed a greater *IGF1R* expression compared with wild type cases (p = 0.002). In addition, high *IGF1R* expression was not significantly different comparing patients with low and high *NOTCH1* mutation burden (data not shown). *IGF1R* was significantly higher in *NOTCH1* mutated patients, either with or without trisomy 12, and in *NOTCH1* wt patients with trisomy 12, than in *NOTCH1* wt cases without trisomy12 patients (p = 0.0035, p = 0.0031 and p = 0.0002 respectively); no differences in *IGF1R* expression levels were observed comparing any of the *NOTCH1* wt without trisomy 12 counterpart groups (Fig. 3). The correlation between *NOTCH1* and *IGF1R* was also confirmed considering CLL patients only (p = 0.001), conversely, among cMBL the association between *IGF1R* expression and *NOTCH1* mutation did not reach statistical significance, although a partial trend was demonstrated (data not shown).

**Fig 4:**
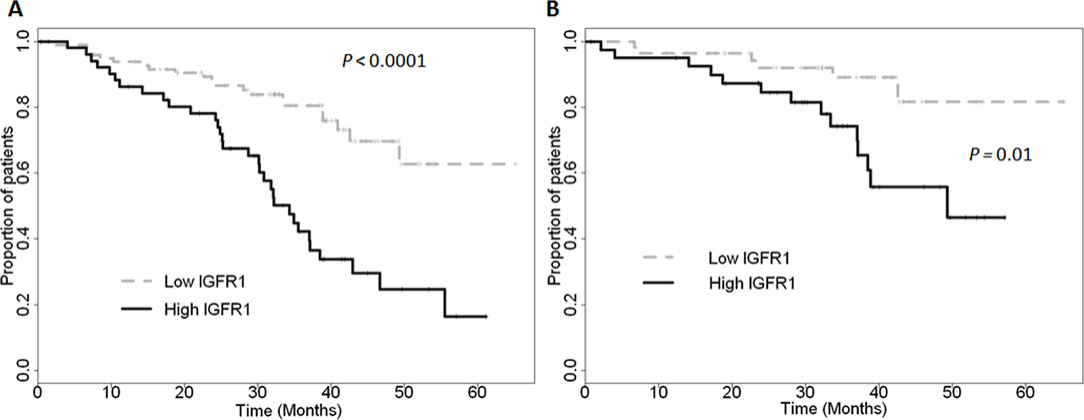
Kaplan-Meier estimated curves of time to first treatment according to *IGF1R* expression considering all CLL patients (A) and only *IGHV*-M patients (B).

Finally, in CLL patients (cMBL not included), increased *IGF1R* expression level was strongly associated with aggressive clinical course and shorter TTFT (Fig. 4A).

Interestingly, *IGF1R* expression was also correlated with disease aggressiveness among *IGHV*-M patients (Fig. 4B). Interestingly, none of the biological prognostic factors (i.e CD38, ZAP-70, FISH) were able to predict clinical outcome in this group. Cox’s proportional hazard model built including the most important biological variables revealed that *IGF1R* expression represented an independent prognostic factor of TTFT in early stage CLL considering either all CLL patients (Table 2) or only *IGHV*-M (Table 3). Among cMBL, *IGF1R* did not show a significant association with TTFT.

**Table 2:** Cox multivariate analysis results considering only CLL patients.

Covariate	p	HR	95% CI of HR
**CD38** **>** **20%**	0.1037	1.6591	0.9045 to 3.0434
**del(11)(q23) and del(17)(p13)**	0.0783	1.8636	0.9353 to 3.7132
***IGF1R* expression**	0.0258	1.0033	1.0004 to 1.0062
***IGHV* mutational status**	0.0128	2.3390	1.2019 to 4.5519

HR: hazard ratio. CI: confidence interval.

**Table 3:** Cox multivariate analysis results considering only IGHV-M CLL patients.

Covariate	p	HR	95% CI of HR
**CD38** **>** **20%**	0.070	1.3253	0,3075 to 5,7119
**del(11)(q23) and del(17)(p13)**	0.1446	4.5572	0,6002 to 34,5990
***IGF1R* expression**	0.0094	1.0043	1,0011 to 1,0075

HR: hazard ratio. CI: confidence interval.

## Discussion

*IGF1R* gene is known to regulate normal cell growth and contribute to transformation and proliferation of malignant cells in many cell contexts [6]. This gene has been reported to play an important role in haematological malignancies such as multiple myeloma, mantle cell lymphoma and T-cell acute lymphoblastic leukaemia (T-ALL) [7–9,21]. *IGF1R* was associated also with favorable prognosis in breast cancer, non small cell lung cancer, soft tissue sarcoma and classical Hodgkin’s lymphoma [22–27].

In CLL, it was recently reported that IGF1R is involved in the activation of phosphorylation cascades such as Ras/Raf/MAPK and PI3K-Akt [28].These pathways are emerging as important survival and proliferation signals in CLL and represent an attractive target of novel drugs (i.e ibrutinib and idelasilib) [29–31]. In concordance with a previous study [11], we report the strong association of *IGF1R* with unfavorable/intermediate cytogenetic aberrations, mainly trisomy 12. This association seemed independent of *IGHV*-UM, *NOTCH1* mutation or cMBL status. Based on this data *IGF1R* could represent a potential novel candidate for specific targeted therapy in this cluster of patients.

Our data showed a strong association between *IGF1R* up-regulation and other unfavorable biological features such as *IGHV*-UM and CD38 expression, differently from what reported by Yaktapour et al [11]. In agreement with these biological associations, high *IGF1R* expression was independently correlated with shorter TTFT among CLL patients and this association retained its significance also considering only *IGHV*-M cases. Interestingly *IGF1R* did not showed a unique association with the most common unfavorable prognostic factors, but its high expression emerged both within trisomy 12 patients, known to have a intermediate clinical course, and *IGHV*-M cases or other apparently favorable subgroups (i.e del13q14 as single lesion) [3–5]. In fact, *IGHV*-M patients are generally characterized by indolent clinical course, even if a significant fraction of these patients progressed and required treatment. To date, no prognostic factor has proven to predict effectively clinical aggressiveness in these patients, and this observation was confirmed in our data set where no standard biological and clinical features were able to predict clinical outcome in terms of TTFT among early stage *IGHV*-M CLL patients (data not shown). In this context, *IGF1R* might represent a potentially useful prognostic marker of TTFT among patients with theoretically indolent biological (*IGHV*-M) and favorable clinical profile at diagnosis (Binet stage A). This suggestion is reinforced by the evidence that *IGF1R* retained its significant correlation in multivariate analysis. Among cMBL, *IGF1R* expression could be an important indicator of disease aggressiveness albeit this needs to be validated in larger cohort with longer follow up.

*IGF1R* gene and protein levels and activity were recently reported to be strongly associated with the NOTCH1 pathway in patients affected by T-ALL, in which it is frequently involved and activated by mutations or molecular aberrations [7]. Activating *NOTCH1* mutations have recently been reported by many groups as one of the most frequent aberrations in refractory and transformed CLL [32–35]; *NOTCH1* c.7541_7542delCT allelic variant represents the most frequent aberration, accounting for more than 80% of all mutations [18,32–35]. According to *NOTCH1* mutation data in our cohort, we observed that patients harboring *NOTCH1* c.7541_7542delCT mutation were characterized by a significant *IGF1R* up-regulation. Thus, it may suggest the existence of potentially altered signalling pathway, such as *IGF1R*, in CLL patients harboring *NOTCH1* mutation. Based on these data, *IGF1R* activity may provide an important growth and survival advantage to CLL cells, similar to what has recently been reported in T-ALL [7].

Trisomy 12 was historically described as intermediate cytogenetic aberration characterized by heterogeneous clinical course [3]. This was confirmed also in our prospective series where a fraction of trisomy 12 patients showed an indolent clinical course, whereas the others had a more aggressive evolution and short TTFT. This latter group was generally characterized by *IGHV*-UM and/or high CD38, *NOTCH1* mutation, high *IGF1R*. In fact, patients with trisomy 12 showing low *IGF1R* expression, and *IGHV*-M and *NOTCH1* wt status were generally characterized by indolent clinical course. These data strengthen the potential role of *IGF1R* to discriminate aggressive entities among “apparently intermediate” and/or “favorable CLL”.

Recent improvements in gene sequencing have demonstrated the importance of subclones in cancer development. As reported in our previous study, *NOTCH1* mutation was defined as subclonal in all patients with positive ARMS-PCR and negative by Sanger sequencing [18]. All these patients were characterized by a mutation allele burden between 0.7 and 7%. Comparing subclonal vs. clonal mutated *NOTCH1* patients, no significant clinical or biological difference was observed [18], and this was true also for *IGF1R* expression (data not shown). The potential prognostic and biological role of subclonal gene mutations in CLL is a matter of debate [18,36,37]; available data suggest that clonal and subclonal mutations share common clinical and biological features, thus suggesting a potential perturbation of the same signalling pathways.

Overall, our study shows the importance of *IGF1R* expression in CLL and its strong association with specific adverse clinical and biological features, confirming the interest for the study of this gene as a potential prognostic factor and its possible role as a therapeutic target in a specific group of CLL patients carrying trisomy 12 and *NOTCH1* mutations.

## Supporting Information

S1 Fig Q-RT-PCR validation of the microarray expression data. The correlation coefficients of expression levels were assessed for all transcripts and are shown in the chart. Both the microarray and Q-RT-PCR data have been scaled in the range 0–1.
(TIF)

S2 Fig Boxplots of *IGF1R* expression levels in subset #4, *IGHV*-UM and *IGHV*-M patients. (TIF)

S1 Table Most common stereotyped subsets prevalence and corresponging IGF1R expression metrics. (DOCX)
