# Supplementary figures and images for: Insulin Growth Factor 1 Receptor Expression Is Associated with NOTCH1 Mutation, Trisomy 12 and Aggressive Clinical Course in Chronic Lymphocytic Leukaemia
Source: PLoS One. 2015 Mar 18;10(3):e0118801. doi: 10.1371/journal.pone.0118801 (PMC4365018; doi:10.1371/journal.pone.0118801)

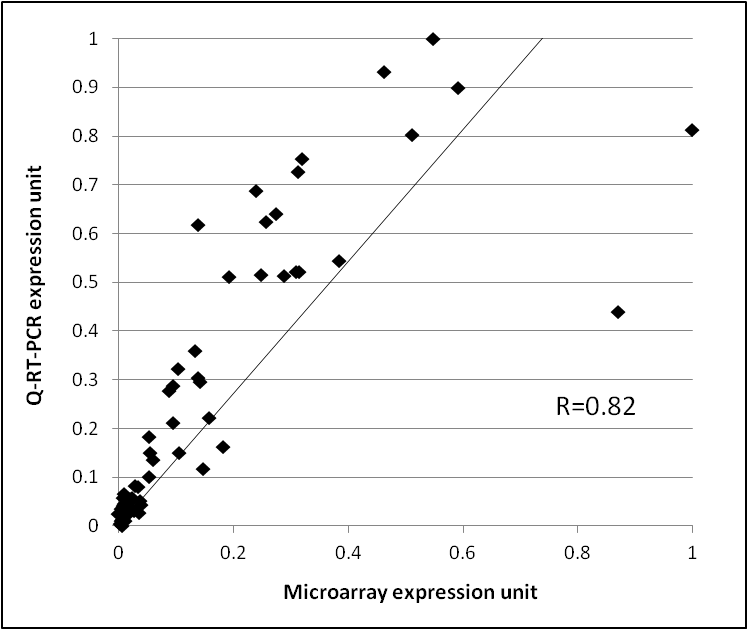

Supplement: S1 Fig — The correlation coefficients of expression levels were assessed for all transcripts and are shown in the chart. Both the microarray and Q-RT-PCR data have been scaled in the range 0–1. (TIF) [file pone.0118801.s001.tif]

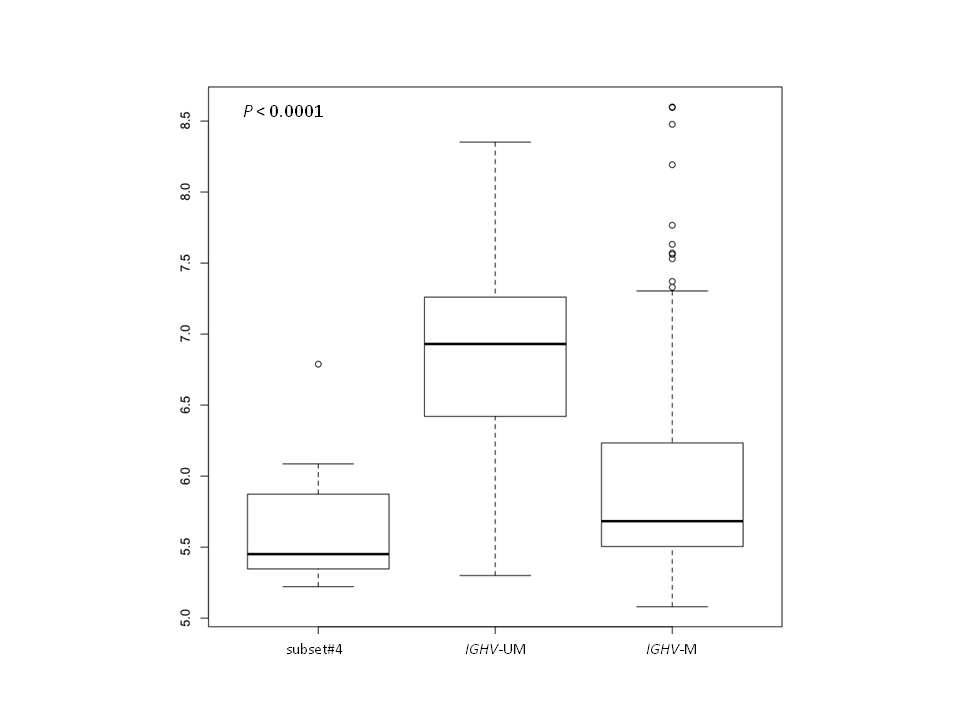

Supplement: S2 Fig — (TIF) [file pone.0118801.s002.tif]
