# Supplementary material for: Insulin Growth Factor 1 Receptor Expression Is Associated with NOTCH1 Mutation, Trisomy 12 and Aggressive Clinical Course in Chronic Lymphocytic Leukaemia
Source: PLoS One. 2015 Mar 18;10(3):e0118801. doi: 10.1371/journal.pone.0118801 (PMC4365018; doi:10.1371/journal.pone.0118801)
Supplement: S1 Table — (DOCX) [file pone.0118801.s003.docx]

**Table S1.** Most common stereotyped subsets prevalence and their median IGF1R expression.

| **Stereotyped HCDR Subset** | **N° Pts** | **Median IGF1R expression (range)** |
| --- | --- | --- |
| Sub #1 | 5 (2,3%) | 6,9 (5,71-7,5) |
| Sub #2 | 2 (0,9%) | 5,989 (5,5-6,4) |
| Sub #3 | 2 (0,9% | 6,98 (6,97-6,99) |
| Sub #4 | 10 (4,5%) | 5,45 (5,22-6,78) |
| Sub #7 | 5 (2,3%) | 7,01 (6,49-7,63) |
| Sub #8 | 3 (1,4%) | 7,09 (5,48-8,35) |
| Sub #9 | 2 (0,9%) | 7,2 (7,08-7,33) |
| Sub #10 | 4 (1,8) | 7,19 (6,67-7,76) |
| Other Sterotyped | 37 (18%) | 6,79 (5,32-8,59) |
| Not sterotyped HCDR | 147 (67%) | 5,86 (5,08-8,59) |
